# Localization of Methyl-Coenzyme M Reductase as Metabolic Marker for Diverse Methanogenic Archaea

**DOI:** 10.1155/2013/920241

**Published:** 2013-02-25

**Authors:** Christoph Wrede, Ulrike Walbaum, Andrea Ducki, Iris Heieren, Michael Hoppert

**Affiliations:** ^1^Institute of Microbiology and Genetics, Georg-August-Universität Göttingen, Grisebachstraße 8, 37077 Göttingen, Germany; ^2^Hannover Medical School, Institute of Functional and Applied Anatomy, Carl-Neuberg-Straße 1, 30625 Hannover, Germany; ^3^Behavioral Ecology and Sociobiology Unit, German Primate Center, Kellnerweg 4, 37077 Göttingen, Germany; ^4^School of Veterinary and Biomedical Sciences, Murdoch University, 90 South Street Murdoch, WA 6150, Australia; ^5^Courant Centre Geobiology, Georg-August-Universität Göttingen, Goldschmidtstraße 3, 37077 Göttingen, Germany

## Abstract

Methyl-Coenzyme M reductase (MCR) as key enzyme for methanogenesis as well as for anaerobic oxidation of methane represents an important metabolic marker for both processes in microbial biofilms. Here, the potential of MCR-specific polyclonal antibodies as metabolic marker in various methanogenic Archaea is shown. For standard growth conditions in laboratory culture, the cytoplasmic localization of the enzyme in *Methanothermobacter marburgensis*, *Methanothermobacter wolfei*, *Methanococcus maripaludis*, *Methanosarcina mazei*, and in anaerobically methane-oxidizing biofilms is demonstrated. Under growth limiting conditions on nickel-depleted media, at low linear growth of cultures, a fraction of 50–70% of the enzyme was localized close to the cytoplasmic membrane, which implies “facultative” membrane association of the enzyme. This feature may be also useful for assessment of growth-limiting conditions in microbial biofilms.

## 1. Introduction

Methyl-coenzyme M reductase (MCR) is the key enzyme of the final, methane-forming step in methanogenesis. The enzyme catalyses the reductive cleavage of methyl-coenzyme M (CoM-S-CH_3_) using coenzyme B (HS-CoB) as reductant which results in the production of methane and the heterodisulfide CoM-S-S-CoB. Though the involved enzyme complexes as well as the reactants differ between *Methanosarcinales*, *Methanobacteriales,* and other groups of methanogens, the essential reaction steps are similar and require several membrane-dependent steps (see [1, 2] for review). The formation of methyl-coenzyme M is catalysed by one subunit (MtrE) of a membrane-bound complex (the N^5^-methyl-tetrahydromethanopterin:coenzyme M methyltransferase) and is coupled with energy conservation via an electrochemical sodium potential across the cytoplasmic membrane (see [3] for review). Regeneration of the reductant HS-CoB is brought about by the enzyme heterodisulfide reductase. For the regeneration of HS-CoB, reducing equivalents are needed, provided by hydrogenases and/or dehydrogenases. The reducing equivalents are either guided via a membrane-bound electron transport chain to the enzyme or are directly transferred from the hydrogenase to the heterodisulfide reductase. The reactions are also coupled to chemiosmotic mechanisms, resulting in the generation of ATP via a H^+^-potential [4–6]. Like MtrE, the heterodisulfide reductase is a part of a membrane-bound complex. The methyl-coenzyme M reductase reaction step itself is not membrane-dependent. The enzyme has been purified from the cytoplasmic fractions of methanogenic Archaea and has been localized in the cytoplasm by immunoelectron microscopy. The catalytic reaction does not depend on the addition of membrane preparations [7–11]. A number of experiments, however, indicate that there is a certain affinity of the enzyme to the membrane [12, 13]. MCR of *Methanothermobacter marburgensis* was located at the cytoplasmic membrane under nickel-depleted growth conditions. Also electron microscopy of vesicle preparations from *Methanobacteriales* and *Methanosarcina* showed that at least a fraction of MCR is membrane-associated. From these data, it was deduced that MCR might be part of a membrane-bound multienzyme complex [14, 15].

For the reverse process, the anaerobic oxidation of methane, a reverse operating methanogenic pathway has been postulated, with an MCR structurally very similar to the canonical enzyme [16–18]. In the postulated pathway, again, membrane binding is not necessarily required. However, as in methanogenesis, membrane association might also be of advantage, since the same membrane-dependent processes as in methanogenesis are likely [17, 19].

In *Methanobacterium thermoautotrophicum*, two different localizations of the MCR could already be shown [13]. In our study, we show that these results are also true for other methanogens, and we will discuss these results in view of immunolocalization of the key enzyme MCR for studies in environmental biofilms.

## 2. Materials and Methods

**Figure 1:**
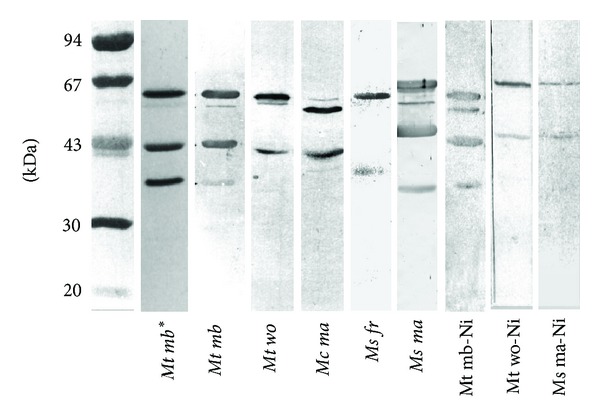
Specificity of the polyclonal serum used for immunolocalization. The slots depict crude extracts of the organisms after Western blotting of SDS gels and double-immunoperoxidase precipitation. All slots show the typical pattern of MCR. For most organisms (except *Ms ma* and *Mt mb*), only the two larger of the three MCR subunits are visible. When cells were grown on nickel-depleted media, the respective slot is marked with -Ni. The second slot (marked with an asterisk) shows a silver stained SDS-polyacrylamide gel of the purified enzyme. *Mt mb: Methanothermobacter marburgensis*, *Mt wo: Methanothermobacter wolfei*, *Mc ma: Methanococcus maripaludis*, *Ms fr: Methanosarcina mazei* (DSM 3318, formerly *Methanosarcina frisia*), *Ms ma: Methanosarcina mazei* (DSM 3647).

*Methanothermobacter marburgensis* (DSM 2133, formerly *Methanobacterium thermoautotrophicum*, strain Marburg), *Methanothermobacter wolfei* (DSM 2970, formerly *Methanobacterium wolfei*), and *Methanococcus maripaludis* (DSM 2067) were grown autotrophically as described [20–23]. *Methanosarcina mazei* (DSM 3318, formerly *Methanosarcina frisia*) and *Methanosarcina mazei* (DSM 3647) were grown heterotrophically [24, 25]. Nickel-limited media did not contain nickel salts in trace element solutions and were supplemented with up to 200 mM levulinic acid (cf.  Table 1). For immunolocalization, cells were grown in batch cultures at linear growth rates with approximate doubling times between 25 and 45 h (Table 1). Cell disruption was performed with a French pressure cell operated at 1,500 lb/in^2^ and subsequent centrifugation by 15,000 ×g for 25 min at 4°C in order to remove cell debris. The supernatant was used for Western-blotting (see below). For protein purification, cells of *Methanothermobacter marburgensis* were grown in 14 l-fermenters with a doubling time of 2.9 h in the exponential phase on mineral salt medium and continuous gassing with H_2_/CO_2_ (80%/20%, v/v) as described [20]. Purification of MCR was performed according to [7]. The purified protein (MCR, i.e. the isoform I of methyl-coenzyme M reductase, Figure 1) was used for production of polyclonal antisera [26]. Protein purity and specificity of the antisera was tested by SDS polyacrylamide gel electrophoresis and Western blotting [27–29] and by immunolocalization control experiments (see below, [30]). Protein assays were performed according to [31].

Samples of an environmental methane-oxidizing biofilms were obtained and processed as described [32, 33]. Microbial mat samples were collected in 2001 during a cruise with the Russian R/V “Professor Logachev” from the methane seep area located on the NW' Shelf region (Crimean Shelf) in the Black Sea. Material for transmission electron microscopy and immunofluorescence analyses was chemically fixed in a 4.0% (w/v) formaldehyde solution and kept at 4°C in 100 mM PBS (phosphate-buffered saline, pH 7.0). The samples were washed several times in PBS and fixed in 0.3% (v/v) solution of glutardialdehyde and 0.5% (w/v) formaldehyde in PBS for 2 h at 4°C. The samples were then washed three times in PBS supplemented with 10 mM glycin. See below for subsequent dehydration and resin embedding.

Active cultures were chemically fixed anaerobically by adding 0.2% (v/v) solution of glutardialdehyde and 0.3% (w/v) formaldehyde to the active culture under anaerobic conditions. After incubation for 2 h at 4°C, the culture was centrifuged three times for 10 min at 9.000 ×g and resuspended in PBS supplemented with 10 mM glycin. Molten agar (2%, w/v, 50°C) was added to an equal volume of the resuspended pellet. After mixing thoroughly, the sample was allowed to solidify.

Subsequently, biofilm samples and agar-embedded culture samples were dehydrated. For dehydration, an ascending methanol series was used [30]: 15% (v/v), 30% for 15 min, 50%, 75% for 30 min, 90%, and 100% for 1 h. The temperature was successively lowered down to −35°C (steps: 15%, 30% at 0°C, 50% at −20°C, and all other steps at −35°C). Samples were then incubated in Lowicryl K4M resin dilutions in methanol (1 : 3, 1 : 2, and 3 : 1; Lowicryl resin obtained from Electron Microscopy Sciences, Hatfield, PA, USA), followed by incubation in pure resin for 1 h per step and then overnight. The blocks were transferred to small gelatin capsules (Plano, Wetzlar, Germany) containing pure resin and were polymerized for at least 48 h at −35°C and 3 d at room temperature under UV light.

Ultrathin sections of trimmed specimens (80–90 nm) were cut with glass knives in a Reichert Jung FC 4 ultramicrotome (Leica Microsystems, Wetzlar, Germany). Sections were transferred onto Formvar-coated grids [30].

For TEM immunocytochemistry, grids were placed with sections facing downwards, for 30 min on drops of 3% (w/v) bovine serum albumin (BSA) in PBS, then for 2 h on anti-MCR antibodies (1 mg protein/mL; dilution in PBS). Negative controls were performed by incubation of the grids on PBS without the antibody.

Grids were then washed by incubation (two times 5 min) on drops of PBS containing 0.05% (v/v) Tween 20 and by incubation (5 min) on PBS without Tween, followed by a 1 h incubation step on the secondary antibody (goat anti-rabbit IgG-10 nm gold conjugate; British Biocell International Ltd., Cardiff, UK). The secondary antibody was used in a 1 : 80 dilution (in the same solutions as used for the respective primary antibodies). Again, two 5 min washing steps with PBS containing 0.05% (v/v) Tween 20, and one step with PBS was performed, followed by washing for 10 s in distilled water for desalting. Poststaining was performed with 4% (w/v) uranyl acetate solution for 3 min. All steps were conducted at room temperature.

**Figure 2:**
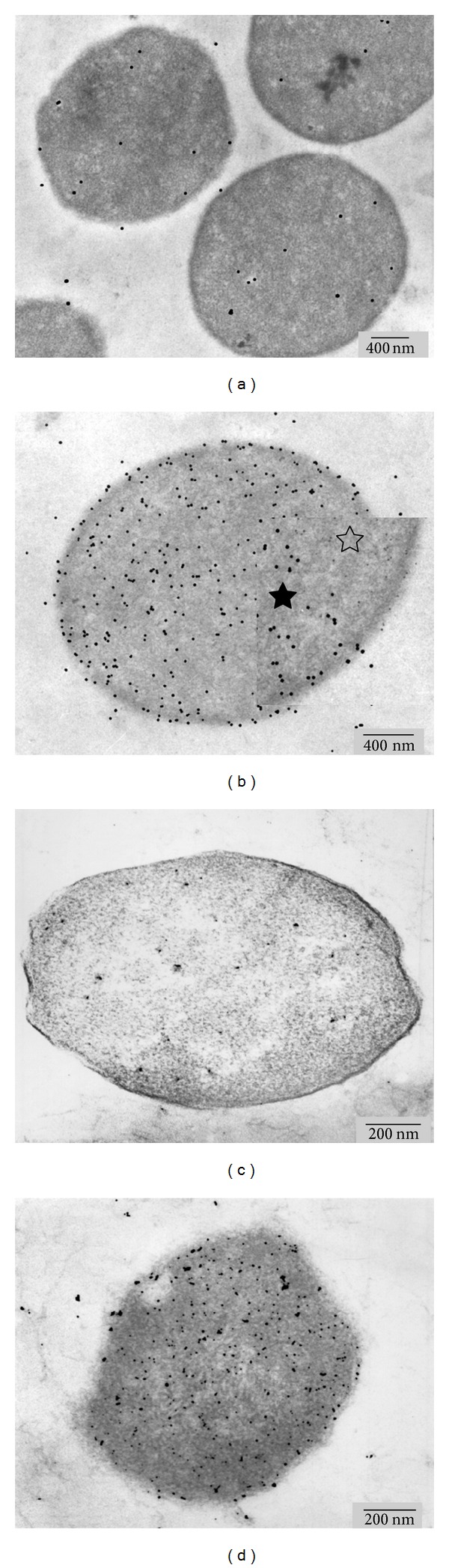
Ultrathin sections of *Methanosarcina mazei *(DSM 3318, formerly *Methanosarcina frisia *(a, b) and *Methanococcus maripaludis* (c, d) grown on media without nickel depletion. Immunolabeled cells (b, d; a, c are negative controls) show cytoplasmic localization of MCR. The inset in (b) shows an enlarged area of the image before (open star symbol) and after processing (closed star) of the gold marker.

Electron micrographs were taken, at calibrated magnifications, with a Philips EM 301 transmission electron microscope (Philips, Eindhoven, The Netherlands) operated in the conventional bright field mode and a Jeol JEM 1011 (Jeol, Eching, Germany) equipped with a Gatan Orius SC1000 CCD camera (Gatan, Munich, Germany). For enhancement of gold particles of 5 nm in diameter, images were processed as follows. High-pass filtering was applied to suppress low spatial frequencies, that is, large image components with smooth contrast gradients. After readjustment of the contrast level by stretching the intensity histogram, the image was transformed by thresholding at a gray level of 100. In the black and white image, particles larger than 7 nm and smaller than 4 nm (original size) were eliminated. The resulting images depict 5 nm gold particles in pure black and white contrast. Particle sizes were enlarged by a factor of 2.5 to allow an easier identification. When these images were merged with the original, a higher final contrast was gained (Figure 2(b), inset). Image processing was performed with the NIH Image software (National Institute of Health; see also [30, 34]). For statistical analysis, 20 randomly selected cells were counted. Gold markers in a range of 25 nm (original size) inside or outside the cytoplasmic membrane were referred to as “membrane associated”; all other markers inside the cell were referred to as cytoplasmic.

## 3. Results and Discussion

The apparent subunit molecular weights (*α*: 65 kDa, *β*: 49 kDa, and *γ*: 38 kDa) of the purified methyl-coenzyme M reductase correspond to the subunit molecular weights of the MCR I isoenzyme from *Methanothermobacter marburgensis* [35]. This isoform is predominant when H_2_ and CO_2_ supply is low and growth limiting [11]. Western blotting of crude extracts, prepared from cells that were also used for immunolocalization, revealed that the antiserum detects protein bands from a variety methanogens. The apparent sizes of these bands correspond to the expected molecular weights of the *α* and *β* subunit of MCR. In some cases, also the smallest (*γ*) subunit is visible (Figure 1). The same pattern has already been shown for MCR extracts obtained from anaerobic methane-oxidizing microbial mats [36]. The *γ* subunit of MCR is known to be less immunogenic and produces a weaker or no signal (Figure 1; [8, 15]). In some of the Western blots, a second band in the range of 67 kDa may account for the presence of the MCR isoenzyme II (MRT) in minor amounts. This enzyme exhibits a slightly smaller *α* subunit and a *γ* subunit of 33 kDa [35]. MRT was isolated from *Methanothermobacter marburgensis *[35] and from various other *Methanobacteriales* and *Methanococcales*, but as yet not from *Methanosarcinaceae* [35, 37–40]. The hydrogenotrophic methanogens investigated here were grown in batch culture with limited substrate availability. Thus, it may be expected that for those methanogens that contain, in analogy to *Methanothermobacter marburgensis*, two methyl-coenzyme M reductases, the MCR isoenzyme I is dominant [11]. 

**Figure 3:**
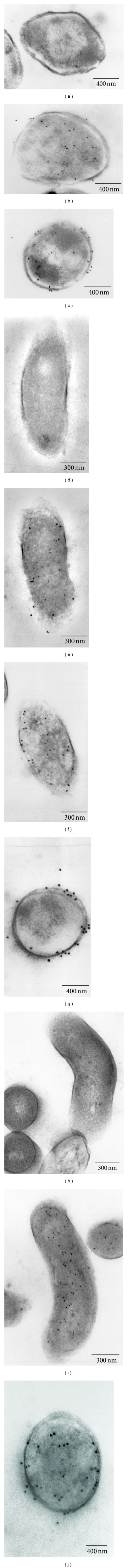
Localization of MCR in *Methanosarcina mazei* (DSM 3647; a, b, c), *Methanothermobacter marburgensis* (d–g), and *Methanothermobacter wolfei* (h, i, j). On media without nickel depletion, the markers are localized in the cytoplasm (b, e, i). On nickel-depleted media with levulinic acid (c, f: 0.05 M; g 0.2 M; j: 0.1 M levulinic acid) a tendency to membrane localization is obvious (a, d, h are negative controls).

Immunolocalization of MCR from *Methanosarcina mazei* (DSM 3318, formerly *Methanosarcina frisia*; Figure 2(b)), *Methanococcus maripaludis* (Figure 2(d)), *Methanosarcina mazei* (DSM 3647; Figure 3(b)), *Methanothermobacter marburgensis* (Figure 3(e)), and *Methanothermobacter wolfei* (Figure 3(i)) grown on media without depletion of nickel show that MCR antigens are distributed throughout the whole cell. For the autotrophically growing *Methanothermococcus thermolithotrophicus* (DSM 2095, formerly *Methanococcus thermolithotrophicus*) and for *Methanolobus tindarius* (DSM 2278), cytoplasmic localization of MCR has already been shown [33]. Though most of the organisms tested did not grow on nickel-depleted media, *Methanosarcina mazei *(DSM 3647), *Methanothermobacter marburgensis,* and *Methanothermobacter wolfei* grew on media without the trace element nickel and after addition of up to 0.2 M levulinic acid (final concentration) to the respective standard growth medium. Levulinic acid inhibits the biosynthesis of the nickel tetrapyrrole cofactor F430. This reduces, in addition to nickel-limitation, the expression of MCR [13, 41]. Under these conditions, the distribution of the enzyme changed; a higher amount of MCR is now located at the membrane.  Table 1 summarizes the results obtained after immunolocalization. Statistical errors of individual cells counted were around 20% for all counts. Thus, the values show a clear trend, but not an exactly reproducible value. Cellular redistribution of MCR markers was most pronounced in *Methanothermobacter marburgensis* (Figures 3(f) and 3(g)). The organism may be considered as a reference for our experiments, since similar results have been described previously [13]. Also *M. wolfei* and *M. mazei* (DSM 3647) showed redistribution of the gold marker (Figures 3(c) and 3(j)). Though the effect is less obvious than in *M. marburgensis*; 50–60% of the markers could be located at the membrane.

**Figure 4:**
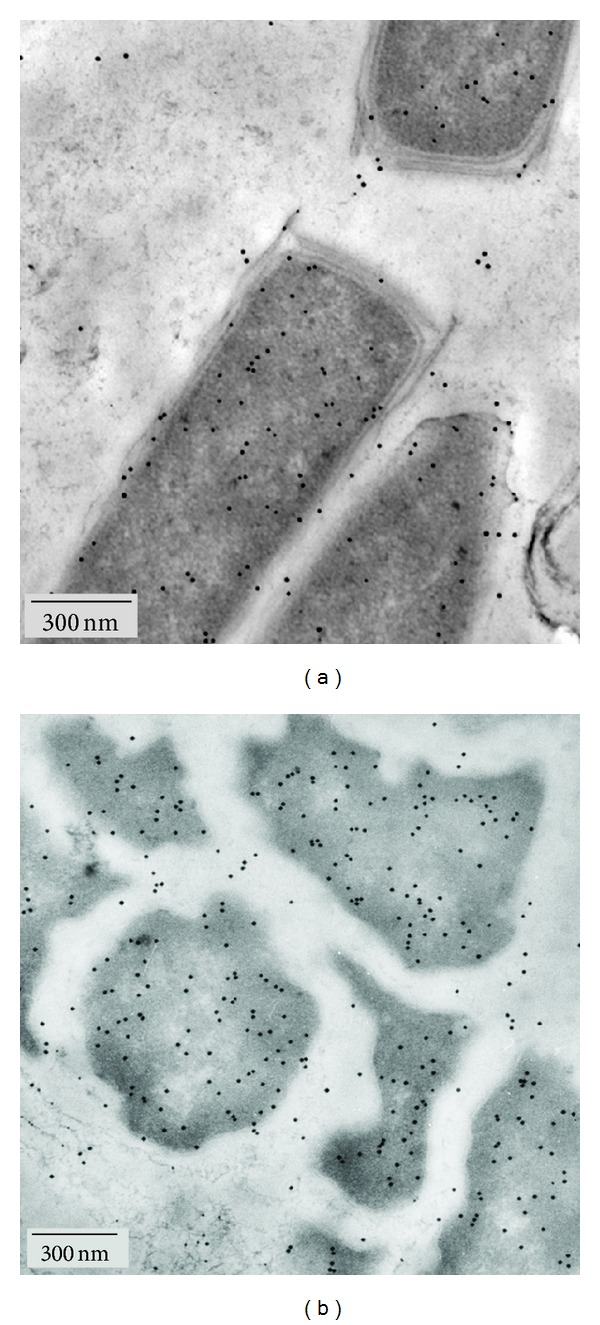
Localization of MCR in predominant morphotypes of microbial mats conducting AOM. Filamentous ANME-1 in the pink layer of the AOM community (a) and coccoid ANME-2 in the black layer (b) exhibit dense cytosolic MCR labelling.

Samples taken from different layers of the multilayered anaerobically methane-oxidizing microbial mats [32, 36] show distinct mophotypes of methane oxidizing Archaea [33]: ANME-1 Archaea are filamentous organisms, related to *Methanomicrobiales* and are dominating in the pink-coloured layer of the microbial mat, whereas the ANME-2 Archaea of the outermost black layer are related to *Methanosarcinales* [42, 43]. Immunolocalization of MCR showed for both morphotypes intensive cytosolic labelling (Figures 4(a) and 4(b)). In this respect, the expression of MCR may not be limited in the environmental biofilm. 

According to all available biochemical data, membrane association of MCR is not necessary for functioning. However, localization of the soluble enzyme in vicinity to the membrane is favourable for the whole pathway, in particular when the enzyme production is limited. Membrane binding becomes obvious, as already stated before [13], when growth conditions limit the synthesis of MCR. Putatively, the diffusion paths of the reactants are shorter and the final step of methanogenesis is more effective. We could show that this feature is not restricted to *M. marburgensis,* but appears to be true for the related *M. wolfei* as well as the phylogenetically distant* M. mazei* and may also be expected for the related methane-oxidizing Archaea. Thus the enzyme may be an interesting example for a “facultative” membrane association of proteins, with a certain capability of membrane binding, but without a specific membrane-dependent metabolic mechanism [44]. For environmental processes, location of the enzyme at the membrane may be an indirect indicator for the physiological status, in our case for nickel-limited conditions and reduced cofactor biosynthesis. According to this assumption, this does not appear to be the case for ANME-1 and ANME-2, prominent in AOM-performing microbial mats from the Black Sea (cf. Figures 4(a) and 4(b)). The images show dense cytoplasmic localization of the marker, accounting for nonlimited production of the enzyme.

## Figures and Tables

**Table 1:** Partitioning of MCR as revealed by immunolocalization.

Organism	Approximate doubling time (h)	Concentration of levulinic acid in the medium	% markers at the membrane
*Methanothermobacter marburgensis *	26	0.0	17
	35	0.05	36
	38	0.2	70
*Methanothermobacter wolfei *	34	0.0	15
	42	0.1	52
*Methanosarcina mazei* (DSM 3647)	20	0.0	32
	34	0.05	60
